# Bilateral Deficit in Countermovement Jump and Its Influence on Linear Sprinting, Jumping, and Change of Direction Ability in Volleyball Players

**DOI:** 10.3389/fphys.2022.768906

**Published:** 2022-02-02

**Authors:** Jernej Pleša, Žiga Kozinc, Nejc Šarabon

**Affiliations:** ^1^Faculty of Health Sciences, University of Primorska, Izola, Slovenia; ^2^Andrej Marušič Institute, University of Primorska, Koper, Slovenia; ^3^Human Health Department, InnoRenew CoE, Izola, Slovenia; ^4^Laboratory for Motor Control and Motor Behavior, S2P, Science to Practice, Ltd., Ljubljana, Slovenia

**Keywords:** bilateral deficit, agility, vertical jump, 505 test, approach jump

## Abstract

We investigated the association between bilateral deficit (BLD) in the countermovement jump (CMJ) and change of direction (CoD) performance, CoD deficit, linear sprint, and approach jumping task. The participants (47 young volleyball players; age: 20.8 ± 3.8 years) performed bilateral and single-leg CMJs, modified *T*-test, 505 CoD test, 25-m sprints (with 5, 10, and 15 splits), and vertical approach jumps. The CoD deficit was also calculated from the 505 test and 10 m split time. BLD was calculated from CMJ jump height, peak power, and phase-specific force impulses (FIs). Several small to moderate statistically significant correlations (*r* = 0.42–0.49) were found between BLD and 505 times (7 correlations), sprint times (4 correlations), CoD deficit (1 correlation), and approach jump (1 correlation). *T*-test performance was not correlated with BLD variables (*r* = −0.15–0.22). The direction of the correlations indicated that the larger BLD is associated with superior performance, with the exception of 1 correlation for 505 times for the left leg and 1 for CoD deficit for the left leg. However, these two variables showed unacceptable reliability. Our results suggest that BLD could be useful in making decisions about the amount inclusion of unilateral training for volleyball players.

## Introduction

The term bilateral deficit (BLD) is describing the observation that muscle force generated during maximal bilateral actions is lower than the sum of forces of the left and right limb generated during unilateral contractions (Škarabot et al., 2016). The BLD has been the subject of extensive research (Jakobi and Chilibeck, 2001; Škarabot et al., 2016), and several factors and underlying mechanisms of BLD have been suggested [for review, refer to Škarabot et al. (2016)]. Most often, the BLD has been assessed through isometric or dynamic single-joint contraction; however, it is also observed during ballistic actions, such as various types of vertical jumps (Soest et al., 1985; Bobbert et al., 2006; Pain, 2014). The BLD in single-joint tasks is currently attributed to neural mechanisms (Škarabot et al., 2016), such as interhemispheric inhibition, while a substantial proportion of the variance in BLD in jumping has been explained by mechanics (Bobbert et al., 2006). During bilateral jumps, the muscles shorten with higher velocity, which means that the force output is inevitably lower, which also translates into less joint work per leg (Bobbert et al., 2006). While the majority of the studies aimed to explain the underlying mechanisms of BLD, its potential relevance for athletic performance has been somewhat neglected. Previous studies have demonstrated that resistance training emphasizing bilateral actions decreases the BLD, and the training involving unilateral exercises increases it (Häkkinen et al., 1996; Taniguchi, 1998; Janzen et al., 2006; Beurskens et al., 2015). BLD seems to be very plastic, namely, an opposite phenomenon, termed bilateral facilitation, has also been observed in bilaterally trained athletes, such as Olympic weightlifters (Howard and Enoka, 1991). Jumping tasks seem particularly promising in regard to performance, as they probably encompass both neural and mechanical aspects of BLD (Bobbert et al., 2006; Škarabot et al., 2016). If BLD is related to sports performance, it could be used to guide training-related decision-making, such as preferential inclusion of bilateral or unilateral exercises into the training programs of athletes.

To date, only five studies have examined the relationship between BLD and athletic performance (Bračič et al., 2010; Bishop et al., 2019; Ascenzi et al., 2020; Kozinc and Šarabon, 2021; Nicholson and Masini, 2021). It was found that the lower BLD in the countermovement jump (CMJ) was related to higher peak forces measured at the rear leg during sprint start (*r* = −0.63) and higher total force impulse (FI) (*r* = −0.55) (Bračič et al., 2010). In other words, sprinters with higher BLD produced lower rear leg forces and lower total FI, suggesting that BLD should be minimized for optimization of sprint start. However, no performance data, such as sprint times, were obtained in this study. Two subsequent studies reported that higher BLD could be positively related to change-of-direction (CoD) ability (Bishop et al., 2019; Kozinc and Šarabon, 2021). Higher BLD could reflect better neuromuscular capacity in unilateral tasks compared with bilateral tasks. Given that CoD tasks consist of a series of unilateral actions, the abovementioned associations seem reasonable. However, in two other studies, no relationship was found between BLD in CMJ and linear sprint performance or CoD performance (Ascenzi et al., 2020; Nicholson and Masini, 2021). Overall, the literature suggests that BLD could be related to athletic performance, especially to CoD tasks. However, two of the previous studies were not conducted on athletes (Bishop et al., 2019; Nicholson and Masini, 2021), and only two studies (Bishop et al., 2019; Ascenzi et al., 2020) included CoD deficit as a metric of performance. It is well known that the performance of the CoD tasks is heavily influenced by linear acceleration and sprinting ability (Nimphius et al., 2016). Recently, the CoD deficit was suggested as an approach to obtain a more isolated measure of CoD ability and limit the effect of the acceleration and linear sprinting ability (Nimphius et al., 2016). However, the relationship between CoD deficit and BLD remains unexplored. Moreover, the relationship between BLD and sport-specific tasks has not been investigated to date, and none of the aforementioned studies have been conducted on volleyball players.

The purpose of this study was to examine the relationship between BLD, derived from CMJ variables, and CoD performance (*T*-test and 505 test), CoD deficit, linear sprinting ability, and approach jump performance, on a sample of young male volleyball players. Volleyball gameplay involves repeated explosive efforts in multiple directions (Hedrick, 2007). In addition, approach jumps are pivotal for attacking actions in volleyball (Forthomme et al., 2005). We hypothesized that larger BLD in CMJ variables would be associated with better CoD performance (i.e., shorter CoD task times and lower CoD deficit) (Bishop et al., 2019; Kozinc and Šarabon, 2021), as well as better sprint and approach jump performance. In addition to jumping height (JH) and peak power (PP), we also used phase-specific (i.e., braking, propulsive, and total positive) FI outcomes for BLD calculation, as specific abilities, such as eccentric strength, which are known to be crucial for sport-specific performance (Chaabene et al., 2018). Considering the particular importance of eccentric strength for CoD performance (Chaabene et al., 2018), we hypothesized that BLD calculated from braking phase FI presents a higher correlation with CoD performance.

## Materials and Methods

### Participants

For this study, we recruited 47 young male volleyball players (age: 20.8 ± 3.8 years; body height: 187.4 ± 7.8 cm; body mass: 80.8 ± 8.8 kg). All the players have been competing in the 1st or 2nd division of the national league. They reported regular participation in training for 10.6 ± 4.6 years and to attend 5.6 ± 1.5 training sessions per week. They also reported to perform full-body resistance exercises regularly. The inclusion criteria were the absence of injuries in the previous 6 months and the absence of any other medical diseases. All participants were thoroughly informed about the experimental procedures and signed informed consent before proceeding with the testing. For underage participants, their parents or legal guardians signed the consent on their behalf. The experiment was approved by the Republic of Slovenia National Medical Ethics Committee (approval number: 0120–99/2018/5) and was conducted in accordance with the Declaration of Helsinki.

### Study Design

This was a cross-sectional study, conducted in a single visit. The participants had been routinely performing the testing procedures; therefore, no familiarization session was conducted. The participants performed a warm-up, consisting of 10 min of light running on an indoor track, 5 min of dynamic stretching, and 5 min of bodyweight resistance exercises (i.e., squats, lunges, and push-ups). Then, they completed (a) assessments of vertical jump on a force plate (bilateral and unilateral CMJ), for the purpose of calculating BLD and (b) performance tests (25-m linear sprint, modified *T*-test, 505 test, and vertical approach jump). The breaks between the tasks were at least 5 min. The order of the performance tasks was randomized. In all tasks, the average of the repetitions was considered for further analyses.

### Countermovement Jump Assessment

The CMJs were performed on a piezoelectric force plate (Kistler, model 9260AA6, Winterthur, Switzerland). The participants performed 2–3 warm-up trials for each jump (bilateral and unilateral). Each jump was performed 2 times (6 repetitions in total), with 1 min recovery between trials. The hands were placed on the hips at all times. The participants were instructed to perform an explosive counter-movement to the self-selected depth and to jump as high as possible. For the unilateral CMJ, the non-tested leg was slightly flexed at the knee and was not allowed to be touching the tested leg and not allowed to swing during the jump. Both legs were tested unilaterally in alternating order.

Ground reaction force data were recorded at a sampling rate of 1,000 Hz. The signals were automatically processed using the software of the manufacturer (MARS, Kistler, Winterthur, Switzerland) by a moving average filter with a 5 ms window. The FI (force multiplied by time) at each time point (1 ms) was divided by the mass of the participant to determine the change in velocity, which was then added to the velocity of the system to compute a new instantaneous velocity. The JH was calculated based on the takeoff velocity. Instantaneous power was calculated as the product of force and velocity, and the PP was considered as one of the outcome variables. In addition to JH and PP, we considered several phase-specific FI outcome (defined as an integral of force with respect to time) metrics in specific phases of the jump. These phases included the unweighting phase, the braking phase, and the propulsion phase. In addition, the total positive FI (the FI in braking and propulsion phase combined) was also calculated. The BLD_*CMJ*_ was calculated using the following equation (Škarabot et al., 2016):


$$BLD\left(\%\right)=\left(100\times \frac{B\,i\,l\,a\,t\,e\,r\,a\,l}{U\,n\,i\,l\,a\,t\,e\,r\,a\,l\,l\,e\,f\,t+U\,n\,i\,l\,a\,t\,e\,r\,a\,l\,r\,i\,g\,h\,t}\right)-100$$

### Change-of-Direction Performance

The change-of-direction (CoD) assessment involved two tests (modified *T*-test and 505 test). For both tests, we used single-beam laser timing gates (Brower Timing Systems, Draper, UT, United States), which were positioned at the level of greater femoral trochanter for each participant and recorded the times to the nearest 0.001 s. The participants began each task 30 cm behind the start line in order to prevent early triggering of the timing gates. First, a modified *T*-test was conducted. In brief, the modified *T*-test maintains the number and the directions of the traditional *T*-test, but with approximately twofold shorter total distance [refer to Sassi et al. (2009) for details]. The participants started the test at their own discretion. Two warm-up repetitions with submaximal effort were performed first, followed by three test repetitions, with 2 min recovery in between. Then, the 505 test was performed, using the same timing gates. The participants were instructed to sprint to a line that was marked 15 m from the start line (with timing gates positioned 10 m from the start line). They were instructed to plant the left or the right foot on the line, turn for 180° and sprint back 5 m through the timing gates again. Three repetitions were performed for each leg in alternating order, with 2 min recovery between the repetitions. In addition to the total times of the 505 test, we calculated the CoD deficit for each leg, by subtracting 10 m sprint times (refer to the “Linear Sprint” section) from the 505 scores (Nimphius et al., 2016).

### Linear Sprint

Using five pairs of timing gates (the same as above), we collected 0–5, 0–10, 0–15, and 0–25 m sprint times. As with the CoD trials, the participants began each sprint 30 cm behind the start line. A standing start was used, and subjects were free to choose their front leg, which was kept constant across repetitions. Subjects were instructed to sprint from the start line through all sets of timing gates as fast as possible. Five trials were performed, and the recovery time between repetitions was set at 2 min. Sprint split times were used as performance indicators, and the 0–10 m time was also used to calculate the CoD deficit (refer to the “Change-of-Direction Performance” section).

### Vertical Jumps With Approach

Vertical jumps with approach were performed at maximum effort, simulating a spike jump in volleyball (Sorenson et al., 2010). First, standing reach was measured with the dominant arm reaching overhead, while participants were facing the wall. Jumping reach was measured with a measurement tape placed on the basketball board. Before each jump, participants marked their fingertips with chalk to more accurately determine the jump reach. The difference between the reach from standing and the jump reach represented the JH. Using a spike approach, the participants jumped for height and touched as high as possible on the measuring tape at the basketball board. Since all participants were well familiar with the test, they were instructed to perform the jumps in a way that they found most convenient, similar to their personal technique during volleyball practice. Each participant performed two warm-up trials and three testing attempts, with 1 min recovery in between. Measurements were recorded to the nearest 1.0 cm.

### Statistical Analysis

Statistical analyses were performed using SPSS (version 25.0, SPSS Inc., Chicago, IL, United States). Descriptive statistics are reported as mean ± SD. Normality of data distribution was verified using Shapiro-Wilk tests. Correlations among BLD variables and performance variables were assessed with Pearson’s correlation coefficients and interpreted as negligible (<0.1), weak (0.1–0.4), moderate (0.4–0.7), strong (0.7–0.9), and very strong (>0.9). Reliability among repetitions was assessed with intra-class correlation coefficients (ICC) (Koo and Li, 2016) and typical error (Hopkins, 2000), expressed as coefficient of variation (CV). The reliability was considered acceptable when the ICC was >0.75 and CV was <10% (Shechtman, 2013). The threshold for statistical significance was set at *p* < 0.05.

## Results

### Descriptive Statistics and Reliability

The descriptive statistics and reliability values for all performance variables are shown in Table 1. It is noted that only data for the left leg for unilateral CMJ are shown, as there were no differences between the legs in terms of reliability. All performance variables had acceptable absolute reliability (CV = 2.09–9.68%). Relative reliability was also acceptable for most performance variables, with the exception of 505 test time for the left side (ICC = 0.68) and COD deficit for the left side (ICC = 0.67). The CMJ variables all showed acceptable relative (ICC = 0.85–0.99) and absolute reliability (CV = 2.0–9.98%).

**TABLE 1:** Descriptive statistics and reliability for all outcome variables.

Task group	Outcome measure	Trial 1		Trial 2		Trial 3		Reliability	
		Mean	SD	Mean	SD	Mean	SD	ICC	CV
Performance indicators	Approach jump (cm)	68.91	16.53	70.21	16.35	69.80	16.65	0.99	2.64
	*T*-test (s)	5.51	0.35	5.47	0.37	5.44	0.38	0.89	2.31
	505 left (s)	2.42	0.21	2.37	0.16	2.38	0.20	0.68	4.56
	505 right (s)	2.37	0.15	2.34	0.18	2.34	0.15	0.78	3.39
	CODD left (s)	0.60	0.16	0.58	0.14	0.59	0.19	0.67	15.5
	CODD right (s)	0.57	0.15	0.53	0.14	0.55	0.15	0.87	9.68
	5 m (s)	1.56	0.10	1.55	0.10	1.55	0.10	0.89	2.19
	10 m (s)	2.27	0.21	2.29	0.15	2.29	0.15	0.81	3.36
	15 m (s)	2.98	0.24	2.99	0.23	2.97	0.20	0.85	2.98
	25 m (s)	4.25	0.36	4.26	0.29	4.27	0.30	0.92	2.09
Bilateral CMJ	Jump height (m)	0.39	0.08	0.39	0.09	/	/	0.97	3.83
	Peak power (W/kg)	55.04	9.13	54.40	9.11	/	/	0.96	3.37
	FI (Breaking)	110.47	21.84	117.99	21.02	/	/	0.85	7.28
	FI (Propulsion)	216.21	38.29	216.25	38.83	/	/	0.99	2.00
	FI (Total positive)	327.82	56.51	335.45	57.24	/	/	0.97	2.81
Unilateral CMJ	Jump height (m)	0.19	0.06	0.19	0.06	/	/	0.96	6.83
	Peak power (W/kg)	35.20	18.69	35.24	18.51	/	/	0.99	4.95
	FI (Breaking)	67.18	18.97	71.89	18.60	/	/	0.87	9.89
	FI (Propulsion)	154.09	46.79	154.61	46.92	/	/	0.99	3.39
	FI (Total positive)	219.58	52.33	225.72	52.30	/	/	0.97	4.18

*FI, force impulse; ICC, intra-class correlation coefficient; CV, coefficient of variance (typical error expressed as a % of mean). Note that only two trials were performed for jumping tasks. In addition, the 4th and 5th repetitions for the sprint trials are omitted on purpose to preserve the coherence of the table.*

### Bilateral Deficit

**FIGURE 1:**
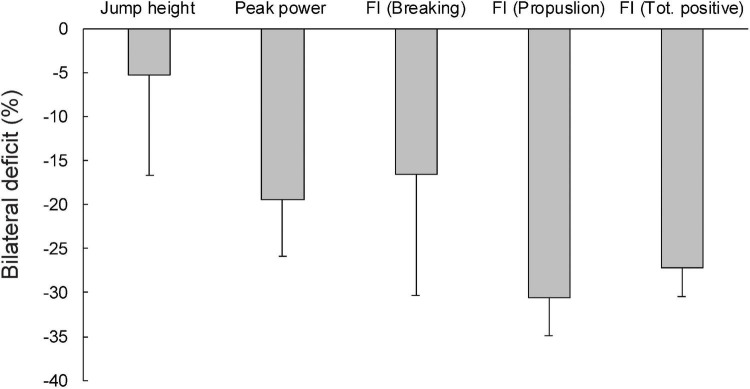
The bilateral deficit in countermovement jump variables. FI, force impulse.

The magnitude of the BLD variables is shown in Figure 1. In addition to the means and SDs, the minimum and maximum values, as well as the number of participants who showed BLD and bilateral facilitation are shown in Table 2. On average, the JH showed the lowest BLD (−5.24 ± 11.44%), with 9 out of 47 participants showing bilateral facilitation. For other variables, the mean BLD was higher, ranging from −30.57 to 16.47% (refer to Table 2 and Figure 1 for details). For the braking phase FI, 5 out of 47 participants showed bilateral facilitation, whereas, for the remaining variables, only 1 participant showed bilateral facilitation.

**TABLE 2:** Descriptive statistics for BLD outcomes.

	Mean	SD	Min	Max	Showing BLD	Showing BLF
BLD jump height	−5.24	11.44	−19.92	39.43	38	9
BLD peak power	−19.46	6.42	−34.14	−4.67	46	1
BLD FI (Breaking)	−16.57	13.82	−36.49	31.88	42	5
BLD FI (Propulsion)	−30.57	4.42	−36.39	−14.95	46	1
BLD FI (Total positive)	−27.13	3.35	−33.49	−21.23	46	1

*BLD, bilateral deficit; BLF, bilateral facilitation; FI, force impulse.*

### Association Between the Bilateral Deficit and Performance Outcomes

The correlations among BLD variables and performance outcomes can be found in Table 3. The approach JH was in moderate negative correlation with BLD in CMJ height (*r* = −0.39; *p* = 0.004). The negative correlation means that the subjects with more BLD (negative number) exhibited higher JHs. Modified *T*-test performance was not correlated with any of the BLD variables (*r* = −0.15–0.22).

**TABLE 3:** Correlations among BLD and performance measures.

Bilateral deficit outcomes	Performance outcomes									
	Approach jump	*T*-test	505 left	505 right	CODD left	CODD right	5 m sprint	10 m sprint	15 m sprint	25 m sprint
BLD jump height	−0.44**	0.22	0.44**	0.36*	0.19	0.18	0.18	0.33*	0.36*	0.49**
BLD peak power	–0.10	0.19	0.41**	0.47**	−0.42**	–0.23	–0.03	0.05	0.11	0.18
BLD FI (Breaking)	–0.05	–0.15	−0.37*	–0.20	0.22	0.21	0.13	0.13	–0.01	–0.15
BLD FI (Propulsion)	–0.30	0.20	0.39*	0.39*	–0.15	0.01	0.00	0.19	0.27	0.42**
BLD FI (Total positive)	–0.12	–0.04	–0.14	0.04	0.19	0.18	–0.05	0.04	–0.01	–0.03

FI, force impulse; BLD, bilateral deficit; CODD, change-of-direction deficit; *p < 0.05; **p < 0.01.

Times to perform the 505 test were in small to moderate positive correlation with BLD for CMJ height (*r* = 0.36–0.44; *p* = 0.003–0.014), peak CMJ power (*r* = 0.41–0.47; *p* = 0.007–0.015), and CMJ propulsion phase FI (*r* = 0.39; *p* = 0.011–0.012). The direction of these correlations also implied that 505 performances improved (i.e., lower times) with higher BLD. On the contrary, a statistically significant negative and small correlation between 505 times for the left leg and BLD for braking phase FI was also present (*r* = −0.37; *p* = 0.017), suggesting that lower BLD in this metric is associated with better 505 test performance. There was a negative moderate correlation between CoD deficit for left leg and BLD in PP (*r* = 0.42; *p* = 0.007), indicating that participants with less BLD had a lower CoD deficit.

The BLD in JH was in small correlation with 10 m sprint time (*r* = 0.33; *p* = 0.032) and 15 m sprint time (*r* = 0.36; *p* = 0.018), as well as in moderate positive correlation with 25 m sprint time (*r* = 0.49; *p* = 0.001). In addition, BLD for propulsion phase FI was in moderate positive correlation with 25 m sprint time (*r* = 0.42; *p* = 0.007). For all correlations related to sprinting, the direction of the coefficient suggests superior performance in players with larger BLD.

## Discussion

The purpose of this study was to investigate the relationship between BLD in CMJ variables and CoD performance (*T*-test and 505 test), CoD deficit, linear sprinting ability, and approach jump performance on a sample of young male volleyball players. The BLD calculated from CMJ height was smaller (mean: −5.24%) compared with BLD in peak CMJ power (mean: −19.46%) and phase-specific FI variables (means: −16.57 to 30.57%). We found that a larger BLD was associated with better performance in linear sprint and approach jump, which was in accordance with our hypothesis. Results for CoD performance were mixed (no association with modified *T*-test and both positive and negative correlations with 505 test and CoD deficit). The BLD derived from the braking phase FI did not show the relationship with performance or showed smaller relationships than BLD calculated from JH. Thus, we rejected our second hypothesis. Although eccentric strength is of paramount importance for sport performance, especially for CoD tasks (Chaabene et al., 2018), this result could be explained by the fact that the CMJ height is a more holistic measure of neuromuscular ability and reflects different types of strength. While CoD tasks are highly dependent on eccentric strength, the evidence has shown that measures of eccentric, concentric, and isometric strength all contribute to CoD performance (Spiteri et al., 2014).

Previous studies have found either no association (Ascenzi et al., 2020; Nicholson and Masini, 2021) or a small to moderate association between BLD in CMJ and CoD performance (Bishop et al., 2019; Kozinc and Šarabon, 2021). The differences among the studies could be partially attributed to different choices of tests, different populations, and sample sizes. Moreover, when interpreting the relationships between BLD and CoD performance, it is important to consider that traditional CoD tests, such as the 505 test (Nimphius et al., 2016), the *T*-test (Križaj, 2020), and the modified *T*-test (Sassi et al., 2009), are correlated with linear sprinting ability. Therefore, it could be that the relationship between BLD and CoD performance was confounded by the relationship between BLD and linear speed ability. The results by Bishop et al. (2019) suggest that higher BLD is not only associated with superior CoD task performance but also with CoD deficit, which is a more isolated measure of CoD ability (Nimphius et al., 2016). In contrast, Ascenzi et al. (2020) reported no such associations. Our results indicated that higher BLD is associated with superior linear sprinting ability, with the correlations increasing with sprint distance. Given that correlations were very small for the first 10 m of a sprint (*r* = 0.18–0.33), it is more likely that the influence of BLD is, in fact, associated with CoD performance, with no or less confounding effect of linear sprint ability. This assumption is well supported by Bishop et al. (2019) who found correlations between BLD and 505 times and between BLD and CoD deficit. The fact that two statistically significant correlations were found in this study, which suggests the opposite relationship between CoD performance and BLD (i.e., larger BLD being detrimental to CoD performance), could be explained by the poor reliability of the CoD task outcomes. These correlations were found for the 505 time and the CoD deficit for the left turn, which was far less reliable (ICC = 0.67–0.68; CV = 4.56–15.5%), compared with the right turn (ICC = 0.78–0.87; CV = 3.39–9.68%). In contrast, it has to be considered that the differences between populations have also contributed to the differences between the studies. Future studies investigating the relationship between BLD and CoD performance on different populations are needed to further clarify this aspect.

We found that larger BLD in CMJ height (3 statistically significant correlations) and FI in the propulsive phase (1 statistically significant correlation) are related to better sprint performance. This is in contrast to previous studies that reported no association between BLD and sprinting performance (Bishop et al., 2019; Ascenzi et al., 2020). However, the directions of the correlation coefficients in a study by Bishop et al. (2019) were the same as in this study, and despite not reaching statistical significance, some coefficients were at least moderate (e.g., *r* = −0.43 for correlation between BLD and 30 m sprint times). The study by Ascenzi et al. (2020) only reported statistical significance without correlation coefficients, making it difficult to compare their results with other studies. In another study, sprinters with a higher BLD were found to produce lower rear leg forces and lower total FI, implying that the BLD should be minimized to optimize sprint start (Bračič et al., 2010). However, given that no performance metric was reported, their study should be interpreted with caution. For now, it could be concluded that BLD in the CMJ may be related to superior linear sprinting, particularly at longer distances; however, the effect is likely small to moderate.

A novel finding of this study was that BLD in CMJ is possibly associated with approach JH. This result is somewhat expected, as the approach jump is performed unilaterally. It has been argued that the BLD should not be necessarily viewed as the deficit, but as “unilateral facilitation” (Škarabot et al., 2016), which can be increased with unilateral training (Häkkinen et al., 1996; Botton et al., 2016). Given the importance of the approach jump task in sports such as volleyball (Forthomme et al., 2005) and basketball, the BLD could be a useful metric to make training-related decisions (e.g., the number of unilateral exercises to be included in the training program). Previous intervention studies comparing the effects of unilateral and bilateral training on strength, power, and speed qualities have reported either no differences between the approaches, or some favorable effects of unilateral training (McCurdy et al., 2005; Botton et al., 2016; Speirs et al., 2016; Gonzalo-Skok et al., 2017; Núñez et al., 2018). It could be that participants with lower BLD (or even bilateral facilitation) are responding faster to unilateral training. Additional research is needed to examine whether lower BLD represents a “window of opportunity” for larger relative improvements in athletic performance in individual athletes. Another consideration for future studies is to incorporate the assessment of the force-velocity relationship when investigating the association between BLD and performance. Namely, the BLD in vertical jumping is largely dependent on the force-velocity relationship of an individual (Bobbert et al., 2006). The force-velocity relationship has been shown to be related to performance, for instance, with spike and serve ball speed in volleyball (Baena-Raya et al., 2021). It could be that the magnitude of the BLD reflects force and velocity capabilities, which are in turn related to performance.

Some limitations and considerations for future research need to be acknowledged and discussed. This study was conducted on male volleyball players, which means that the results should not be generalized to other athletic populations. Moreover, the reliability of the 505 test and the CoD deficit for the left side was unacceptable, which makes the interpretations of these two outcomes difficult. Although the participants were familiar with all testing procedures, additional familiarization sessions could have improved the reliability of CoD outcomes. In addition, although a fair amount of breaks was provided between tasks and repetitions, the overall experimental protocols were relatively demanding, thus, some effects of fatigue cannot be excluded. Finally, although BLD seems to be associated with the performance of volleyball players, it remains to be explored if it is also related to the performance of other sport-specific tasks, such as passing, serving, setting, spiking, blocking, and digging.

## Conclusion

This study found that larger a BLD, calculated from CMJ variables, is associated with superior linear sprint and approach jump performance. Therefore, BLD might be useful as a tool to assist practitioners in decision-making in athletic training. We suggest that a higher amount of unilateral exercises is prescribed for individuals with lower BLD. We recommend using the CMJ height for the calculation of the BLD, as the BLD obtained from the reaming variables showed smaller or no correlation with performance outcomes.

## Data Availability Statement

The raw data supporting the conclusions of this article will be made available by the authors, without undue reservation.

## Ethics Statement

The studies involving human participants were reviewed and approved by the Republic of Slovenia National Medical Ethics Committee. Written informed consent to participate in this study was provided by the participants’ legal guardian/next of kin.

## Author Contributions

JP, ŽK, and NŠ conceptualized the idea. JP and ŽK carried out the measurements and analyzed the data collection. NŠ and ŽK were overviewing the measurement procedures and administration and finalized the manuscript. JP wrote the manuscript. All authors contributed to the article and approved the submitted version.

## Conflict of Interest

NŠ was employed by company S2P, Science to Practice, Ltd. The remaining authors declare that the research was conducted in the absence of any commercial or financial relationships that could be construed as a potential conflict of interest.

## Publisher’s Note

All claims expressed in this article are solely those of the authors and do not necessarily represent those of their affiliated organizations, or those of the publisher, the editors and the reviewers. Any product that may be evaluated in this article, or claim that may be made by its manufacturer, is not guaranteed or endorsed by the publisher.
